# Latent profile analysis of uncertainty in illness and eating self-efficacy in patients with gastric cancer and its associated factors: a cross-sectional study

**DOI:** 10.3389/fpubh.2026.1805637

**Published:** 2026-04-01

**Authors:** Minyi Shi, Chengxi Xia, Yuxin Ye

**Affiliations:** 1 Department of Gastroenterology, Taizhou First People's Hospital, Taizhou, China; 2 Huangyan Hospital, Wenzhou Medical University, Taizhou, China; 3 Tongde Hospital of Zhejiang Province Affiliated to Zhejiang Chinese Medical University (College of Integrated Traditional Chinese and Western Medicine Clinical Medicine), Hangzhou, China

**Keywords:** eating self-efficacy, gastric cancer, health information avoidance, interoceptive sensitivity, uncertainty in illness

## Abstract

**Background:**

Patients with gastric cancer frequently experience substantial psychological burden throughout diagnosis and treatment. Uncertainty in illness and eating self-efficacy are two key determinants of psychological adjustment and rehabilitation. However, prior research has predominantly relied on variable-centered approaches, with limited attention to within-population heterogeneity and its correlates among patients with gastric cancer.

**Objective:**

This study aimed to identify latent profiles of uncertainty in illness and eating self-efficacy among patients with gastric cancer and to examine factors associated with profile membership.

**Methods:**

We conducted a cross-sectional survey and recruited 426 patients with gastric cancer via convenience sampling from two tertiary hospitals in Zhejiang Province, China, between September and December 2025.

**Results:**

Two latent profiles were identified: a Low Uncertainty in Illness–High Eating Self-efficacy profile and a High Uncertainty in Illness–Low Eating Self-efficacy profile. Multivariable logistic regression showed that, using the high uncertainty in illness–low eating self-efficacy class as the reference, health information avoidance, interoceptive sensitivity, male sex, retirement status, lower income, and urban residence were significantly associated with a greater likelihood of belonging to the High Uncertainty in Illness–Low Eating Self-efficacy profile, whereas a history of alcohol consumption was associated with a greater likelihood of belonging to the Low Uncertainty in Illness–High Eating Self-efficacy profile.

**Conclusion:**

These findings suggest that clinicians should deliver stratified, individualized psychological support and nutrition counseling to facilitate patients’ psychological adjustment and rehabilitation.

## 1 Introduction

Gastric cancer is among the most common malignancies worldwide (1, 2). In recent years, advances in early detection and multimodal therapy have improved survival outcomes for some patients (3, 4); nonetheless, the intrinsic complexity of the disease, the protracted treatment course, and persistent uncertainty regarding prognosis and recurrence continue to impose substantial psychological strain (5). A diagnosis of gastric cancer signifies not only physiological disruption but also heightened concerns about disease trajectory (6, 7), doubts about treatment effectiveness, and anxiety regarding future quality of life. Accumulating evidence indicates that psychological factors play a nontrivial role in cancer patients’ illness adaptation, treatment adherence, and recovery (8, 9). Therefore, elucidating the psychological characteristics of this population and their determinants is of considerable theoretical and clinical importance for developing tailored psychological interventions and improving overall quality of life.

Uncertainty in illness refers to the cognitive difficulty individuals experience when constructing meaning from illness-related events (10, 11). Importantly, uncertainty in illness is not merely a negative emotional experience; rather, it is a complex cognitive state shaped jointly by the stimulus frame, cognitive capacity, and structure providers (12). For patients with gastric cancer, sources of uncertainty are multifaceted and layered. On the one hand, tumor heterogeneity across stages leads to substantial variability in treatment selection and prognostic estimation, translating medical complexity into cognitive ambiguity (13, 14). On the other hand, surgery, chemotherapy, targeted therapy, and other modalities entail adverse effects and uncertain efficacy, further intensifying psychological burden (15, 16). Prior studies have linked uncertainty in illness among cancer patients to anxiety, depression, and other negative affective states, and have shown that it can meaningfully influence adaptive coping and quality of life (17, 18). Nonetheless, most existing work treats uncertainty in illness as a unidimensional construct, overlooking the possibility of heterogeneous distribution patterns across patient subgroups—thereby constraining our understanding of psychological diversity in gastric cancer.

Eating self-efficacy denotes an individual’s belief and confidence in their capability to perform specific dietary behaviors (19). Surgery often results in reduced gastric capacity, impaired digestion, and malabsorption, requiring patients to re-establish adaptive dietary patterns (20). In this transition, eating self-efficacy influences whether patients can implement dietary regimens recommended by healthcare teams, thereby affecting nutritional status, weight management, and overall recovery (21, 22). High eating self-efficacy enables patients to maintain proactive coping when confronting dietary difficulties, seek nutritional support, and sustain healthy eating practices; conversely, low eating self-efficacy may engender helplessness and frustration, ultimately undermining needed behavior change (23). Notably, eating self-efficacy is not an isolated psychological construct but is closely intertwined with illness cognition, emotional state, and social support (24). Evidence suggests that cancer patients with lower self-efficacy often report higher uncertainty in illness (18, 25), implying a potential intrinsic linkage between these constructs. However, systematic empirical research examining uncertainty in illness and eating self-efficacy jointly remains limited.

According to stress-and-coping models (26, 27), uncertainty in illness may indirectly shape coping selection and enactment by influencing both primary and secondary appraisal processes (28). When patients experience high uncertainty while processing illness-related information, cognitive ambiguity may erode confidence in behavioral capability, thereby weakening eating self-efficacy (25). From an information-processing perspective (29), elevated uncertainty in illness may indicate difficulties in organizing and integrating health information; the resulting depletion of cognitive resources may spill over into dietary self-management, limiting patients’ ability to use nutritional guidance to build and strengthen eating self-efficacy. Accordingly, we posit that uncertainty in illness and eating self-efficacy may exhibit joint patterning within the gastric cancer population, rather than a simple linear association. Conventional variable-centered approaches are ill-suited to detect such latent categorical structures, underscoring the need for more refined analytic techniques to reveal distinct psychological profiles.

Latent profile analysis (LPA), a person-centered statistical method, classifies individuals into qualitatively distinct latent classes based on response patterns across observed variables (30, 31). Importantly, LPA is a model-based approach that assumes the population may contain unobserved subgroups, making it particularly suitable for detecting hidden heterogeneity in psychological characteristics. In the present study, this is especially relevant because uncertainty in illness and eating self-efficacy may co-occur in different ways across patients with gastric cancer, rather than following a single average pattern. Compared with traditional cluster analysis, LPA is grounded in stronger statistical theory and offers several methodological advantages. Specifically, LPA relies on probabilistic modeling rather than purely distance-based classification, provides objective model-fit indices to determine the optimal number of classes, and estimates posterior probabilities that reflect the uncertainty of class assignment (32). In addition, LPA allows for the inclusion of covariates to examine predictors of class membership, which is highly relevant to the present study given our interest in sociodemographic, clinical, lifestyle, and psychological correlates of latent profile membership. In recent years, LPA has been widely applied in psychology and health sciences to identify subgroups characterized by distinct constellations of psychological and behavioral features—for example, patterns of psychological adaptation, symptom clustering, and quality-of-life trajectories among patients with cancer (33–35).

Despite this progress, several important gaps remain in the current literature:

1. Most existing studies concerning uncertainty in illness and self-efficacy in oncology populations have adopted variable-centered approaches, which emphasize average relationships between variables and may overlook meaningful within-population heterogeneity.
2. Although uncertainty in illness and eating self-efficacy are both highly relevant to the adjustment and rehabilitation of patients with gastric cancer, little is known about whether these two constructs form distinct joint profiles at the individual level.
3. The factors associated with such potential profiles remain unclear, particularly with respect to cognitive and perceptual processes such as health information avoidance and interoceptive sensitivity. This lack of person-centered evidence limits a more precise understanding of psychological heterogeneity in gastric cancer and constrains the development of targeted supportive interventions.

The present study aimed to use LPA to (1) identify latent classes reflecting joint profiles of uncertainty in illness and eating self-efficacy among patients with gastric cancer and (2) determine factors associated with class membership. Specifically, we considered sociodemographic variables, clinical baseline characteristics, lifestyle factors, and psychological variables including health information avoidance and interoceptive sensitivity as potential predictors. Health information avoidance, as a cognitive tendency, reflects motivational avoidance of health-threatening information and may influence uncertainty in illness and eating self-efficacy by restricting access to disease-related knowledge (36). Interoceptive sensitivity refers to individuals’ perception and awareness of internal bodily signals and is particularly relevant for patients with gastric cancer who must monitor bodily responses closely to adjust dietary behaviors (37, 38). Thus, our findings are expected to advance understanding of psychological heterogeneity in gastric cancer and provide evidence to support stratified, individualized psychological and nutritional care in clinical practice.

## 2 Methods

### 2.1 Study design and sample

This cross-sectional study was conducted in the outpatient clinics and inpatient units of the Departments of Medical Oncology and Gastrointestinal Surgery at two tertiary hospitals in Zhejiang Province, China. Participants were recruited by convenience sampling between 12 September and 3 December 2025. Data were collected using an electronic questionnaire administered through the Credamo survey platform.

Prior to recruitment, we coordinated with department heads to obtain support and to identify suitable data-collection periods that would minimize disruption to routine clinical care. During recruitment, trained research staff approached potentially eligible patients in outpatient waiting areas and hospital wards, briefly introduced the study background and aims, and invited participation. Written informed consent was obtained from all participants.

To enhance study rigor, prespecified inclusion and exclusion criteria were applied.

Inclusion criteria were: (1) primary gastric cancer confirmed by endoscopic biopsy and histopathology; (2) aged 18–85 years; (3) able to read and write Chinese sufficiently to understand and complete the questionnaire independently or with assistance from research staff; (4) aware of their gastric cancer diagnosis; (5) clear consciousness and able to communicate effectively; and (6) willingness to participate with provision of written informed consent.

Exclusion criteria were: (1) concurrent primary malignancy at another site or metastatic gastric cancer; (2) history of severe psychiatric disorders (e.g., schizophrenia, bipolar disorder, or major depressive disorder); (3) evident cognitive impairment precluding comprehension of the questionnaire; (4) end-stage disease, unstable vital signs, or current intensive care; (5) receipt of systematic psychotherapy within the past 6 months or concurrent participation in other psychological intervention studies; and (6) inability to complete the survey due to visual, hearing, or other physical impairments.

Latent profile analysis. Simulation work by Nylund-Gibson and Choi (39) indicates that when the sample size exceeds 300, model fit indices in LPA can more accurately recover the true number of classes. In addition, each latent class should include at least 25–50 individuals to ensure stable and reliable estimation of class-specific parameters. Multivariable logistic regression. The minimum required sample size was estimated using G^*^Power 3.1 (40), with *f*^2^ = 0.15, *α* = 0.05, 1 − *β* = 0.95, and 14 predictors, yielding a minimum sample size of 194 participants. Thus, we set the target sample size at no fewer than 400 valid respondents.

Across the two participating hospitals, 536 patients with gastric cancer who met preliminary screening criteria were approached. Of these, 62 were excluded for failing to meet inclusion criteria or meeting exclusion criteria, including 33 outside the age range, 18 with other primary tumors, 11 unaware of their diagnosis, 9 with a psychiatric history, 7 with cognitive impairment, and 4 currently participating in other studies. Among the remaining 474 eligible patients, 48 declined participation, primarily due to lack of interest in the questionnaire, concerns about privacy, or fatigue/physical discomfort. The final analytic sample comprised 426 participants, yielding an effective response rate of 79.47%.

### 2.2 Measures tools

Eating Self-Efficacy Scale. Eating self-efficacy was assessed using the scale developed by Glynn and Ruderman (41), which includes 25 items encompassing two domains: eating in response to negative emotions and eating in socially acceptable contexts. The scale has been applied in Chinese older adults to assess confidence in healthy eating and the ability to differentiate healthy from unhealthy dietary behaviors (42), demonstrating satisfactory cultural applicability and reliability. In this study, items were rated on a 5-point Likert scale ranging from 1 (“no difficulty controlling eating”) to 5 (“extreme difficulty controlling eating”). Total scores range from 25 to 125, with higher scores indicating stronger eating self-efficacy. In the present sample, Cronbach’s *α* was 0.984, indicating good internal consistency.

Mishel Uncertainty in Illness Scale. Uncertainty in illness was measured using the Mishel Uncertainty in Illness Scale (43), designed to assess illness-related uncertainty in adults. A Chinese version translated and validated by Ye, She (44) has demonstrated adequate cultural adaptation and reliability in Chinese patients with malignant tumors. The scale comprises 20 items across three dimensions: ambiguity (8 items), lack of clarity (7 items), and unpredictability (5 items). Responses are scored on a 5-point Likert scale (1 = “strongly disagree” to 5 = “strongly agree”). Total scores range from 20 to 100, with higher scores indicating greater uncertainty in illness. In this study, Cronbach’s *α* was 0.981, indicating good internal consistency.

Health Information Avoidance Scale. Health information avoidance was assessed using the Information Avoidance Scale developed by Howell and Shepperd (45), originally designed to measure the tendency to avoid learning information. The scale includes 8 items and is unidimensional. A Chinese version translated using back-translation by Sun and Wang (46) has shown good cultural applicability and reliability among Chinese older adults. Participants rated each item on a 5-point Likert scale (1 = “strongly disagree” to 5 = “strongly agree”). An example item is: “I would rather not know the dietary requirements for patients with gastric cancer.” Total scores range from 8 to 40, with higher scores indicating stronger health information avoidance. In the present study, Cronbach’s *α* was 0.968, indicating good internal consistency.

Interoceptive Sensitivity scale. Interoceptive sensitivity was measured using the Multidimensional Assessment of Interoceptive Awareness (MAIA) developed by Mehling, Price (47). The MAIA comprises 32 items across eight dimensions: Noticing, Not Distracting, Not Worrying, Attention Regulation, Emotional Awareness, Self-Regulation, Body Listening, and Trusting. A Chinese version translated by Lin, Hsu (48) has demonstrated cultural applicability in Chinese adults. Items were rated on a 5-point Likert scale (1 = “strongly disagree” to 5 = “strongly agree”). An example item is: “When I am tense I notice where the tension is located in my body.” Total scores range from 32 to 160, with higher scores indicating greater interoceptive sensitivity. In this study, Cronbach’s *α* was 0.987, indicating good internal consistency.

### 2.3 Statistical analysis

All analyses were conducted using SPSS 27.0 and Mplus 8.3. Reliability and construct validity were evaluated for each scale using Cronbach’s *α*. In descriptive analyses, continuous variables are presented as means and standard deviations, and categorical variables as frequencies and percentages. Skewness and kurtosis were examined to assess approximate normality. Pearson correlation analyses were performed to evaluate associations among continuous variables and to preliminarily characterize distributions and interrelationships.

Latent profile analysis models were specified using continuous indicator variables. In the primary analysis, all individual item scores from the Mishel Uncertainty in Illness Scale (20 items) and the Eating Self-Efficacy Scale (25 items) were used as indicators, yielding a total of 45 item-level continuous indicators. We applied an iterative model-comparison strategy by fitting models with 1 to 5 latent classes and determining the optimal number of classes based on a comprehensive evaluation of multiple statistical criteria.

Lower values of the Akaike Information Criterion (AIC), Bayesian Information Criterion (BIC), and sample-size adjusted BIC (aBIC) indicate better relative model fit while accounting for model complexity. The Lo–Mendell–Rubin adjusted likelihood ratio test (LMR) and bootstrap likelihood ratio test (BLRT) were used to compare a model with k classes to a model with k − 1 classes; a significant *p* value suggests that the model with one additional class provides a statistically better fit. Entropy was used only as an indicator of classification accuracy, with higher values reflecting clearer separation between profiles, but it was not treated as an index of overall model fit. Final model selection also considered class size and substantive interpretability.

To ensure estimation stability and avoid local maxima, models were estimated using 500 random starting values for the initial stage and 100 optimizations for the final stage; convergence was confirmed by verifying that the best log-likelihood value was replicated across multiple random starts.

Given that the use of 45 item-level indicators increases the number of freely estimated parameters relative to the sample size, we conducted a sensitivity analysis using subscale-level (dimension-level) scores as indicators. Specifically, three subscale scores from the Uncertainty in Illness Scale (ambiguity, lack of clarity, and unpredictability) and two subscale scores from the Eating Self-Efficacy Scale (negative affect eating and socially acceptable eating) were entered as five continuous indicators. The same model-comparison strategy was applied to evaluate whether the optimal class solution and profile characteristics remained consistent with those obtained from the item-level analysis. Methodological simulation research has demonstrated that when latent classes are well separated and class proportions are balanced, sample sizes between 200 and 500 generally provide adequate statistical power to recover two- to three-class solutions reliably, even with a moderate-to-large number of indicators (49, 50).

After selecting the optimal LPA model, independent-samples t tests or one-way ANOVA were used to compare latent profile scores across groups defined by sociodemographic and clinical characteristics.

Subsequently, latent class membership was exported as a categorical variable, and multivariable logistic regression was conducted to identify predictors of class membership. Latent class membership served as the dependent variable, and variables significant in univariable analyses were entered as independent variables. For outcomes with more than two classes, the clinically most relevant or largest class was selected as the reference group. Odds ratios (ORs) and 95% confidence intervals (95% CIs) were calculated for other classes relative to the reference.

### 2.4 Ethical considerations

The study protocol was approved by the Medical Ethics Committee of Taizhou First People’s Hospital. All procedures were conducted in accordance with the Declaration of Helsinki. Written informed consent was obtained from all participants before data collection. Questionnaire data were collected anonymously or in de-identified form and were used solely for research purposes. To protect privacy, questionnaire data were collected anonymously or de-identified and were used only for research purposes.

## 3 Results

### 3.1 Sample characteristics

A total of 426 patients with gastric cancer were included in the analysis. Their sociodemographic, clinical, and lifestyle characteristics are presented in Table 1. Overall, the sample was predominantly male and married, and a considerable proportion reported a history of smoking and alcohol use.

**Table 1:** Sociodemographic and clinical characteristics of the sample.

Variables	Items	Number (*N*)	Proportion (%)
Gender	Male	225	52.8%
	Female	201	47.2%
Education background	Primary school and below	205	48.1%
	Junior high school to high school	146	34.3%
	Bachelor’s degree or above	75	17.6%
Marital status	Divorced	36	8.5%
	Widowed	40	9.4%
	Unmarried	44	10.3%
	Married	306	71.8%
Employment status	Unemployment	47	11.0%
	Retirement	137	32.2%
	Student	18	4.2%
	Employed	224	52.6%
Monthly income level	≤4,000 RMB	177	41.5%
	4,001–8,000 RMB	162	38.0%
	8,001 RMB and above	87	20.4%
Place of residence	Urban	225	52.8%
	Rural area	201	47.2%
TNM stage	Stage I	132	31.0%
	Stage II	173	40.6%
	Stage III	121	28.4%
Smoking	Yes.	281	66.0%
	No.	145	34.0%
Drinking alcohol	Yes.	341	80.0%
	No.	85	20.0%
Age	M ± SD	52.00 ± 18.863	

### 3.2 Descriptive statistics and correlations analysis

Descriptive statistics and correlation coefficients for key variables are shown in Table 2. Mean scores of core variables were all close to the midpoint (*M* = 3.000). Skewness ranged from −0.013 to 0.354 and kurtosis from −0.621 to −0.451, within acceptable limits (|skewness| < 3; |kurtosis| < 8), indicating approximate normality. Correlation analyses showed that uncertainty in illness was strongly negatively correlated with eating self-efficacy. Uncertainty in illness was positively correlated with health information avoidance and interoceptive sensitivity. Eating self-efficacy was negatively correlated with health information avoidance and interoceptive sensitivity. Health information avoidance was positively correlated with interoceptive sensitivity.

**Table 2:** Descriptive statistics and correlations among key variables.

Variables	*M*	SD	Skewness	Kurtosis	1	2	3	4
1. Uncertainty in illness	3.001	0.734	−0.013	−0.579	1			
2. Eating self-efficacy	2.959	0.722	0.012	−0.621	−0.597***	1		
3. Health information avoidance	3.266	0.897	0.354	−0.558	0.227***	−0.290***	1	
4. Interoceptive sensitivity	2.975	0.831	0.169	−0.451	0.224***	−0.266***	0.259***	1

M = Mean; SD = Standard deviation; ***p < 0.001.

### 3.3 Latent profile analysis of uncertainty in illness and eating self-efficacy

Using item-level indicators from the uncertainty in illness and eating self-efficacy measures, LPA models with one to five classes were estimated; fit indices are presented in Table 3. With increasing numbers of classes, AIC, BIC, and aBIC decreased, indicating improved fit. The LMR test indicated that the two-class model fit significantly better than the one-class model (*p* < 0.001), whereas the LMR tests for the three-, four-, and five-class models were not significant (*p* > 0.05). The BLRT was significant for all models (*p* < 0.001). Therefore, the two-class solution was selected as the optimal model.

**Table 3:** Fit indices for LPA models of uncertainty in illness and eating self-efficacy.

Profile	AIC	BIC	aBIC	Entropy	LMR (p)	BLRT (p)	Proportions of potential subgroups
1	48320.323	48685.223	48399.619	–	–	–	–
**2**	**38967.621**	**39519.025**	**39087.447**	**0.984**	**<0.001**	**<0.001**	**53.2%/46.8%**
3	36460.992	37198.900	36621.347	0.971	0.356	<0.001	28.2%/31.8%/40.0%
4	34651.893	35576.305	34852.777	0.977	0.614	<0.001	22.3%/28.0%/28.0%/21.7%
5	33304.850	34415.766	33546.263	0.979	0.239	<0.001	22.1%/14.9%/27.3%/15.3%/20.4%

Bold represents the optimal potential profile.

Model estimation for the two-class solution converged successfully, and the best log-likelihood value was replicated across multiple sets of random starting values, confirming that the solution represented a global rather than a local maximum. Although this ratio is relatively modest, the exceptionally high entropy (0.984) and balanced class proportions (53.2% vs. 46.8%) indicate favorable conditions for stable parameter estimation, consistent with simulation evidence that well-separated classes with balanced proportions can be reliably recovered at this sample size (49).

To further verify the robustness of the class solution, a sensitivity analysis was conducted using five subscale-level indicators (three uncertainty in illness dimensions: ambiguity, lack of clarity, and unpredictability; and two eating self-efficacy dimensions: negative affect eating and socially acceptable eating). Consistent with the primary analysis, the two-class model was supported as the optimal solution. The two-class model showed high entropy (0.903), significant LMR and BLRT results (both *p* < 0.001), and relatively balanced class proportions (46.5 and 53.5%). Although AIC, BIC, and aBIC continued to decrease with additional classes, the LMR test was not significant for the three-class model (*p* = 0.141) or for models with more classes (*p* > 0.05), indicating that more complex class solutions did not provide a statistically superior fit. Critically, the profile characteristics were consistent with those obtained from the item-level analysis: one class was characterized by lower uncertainty in illness and higher eating self-efficacy, whereas the other exhibited the opposite pattern. Class proportions were also comparable. These convergent results across indicator levels support the stability and trustworthiness of the two-class solution.

**Figure 1:**
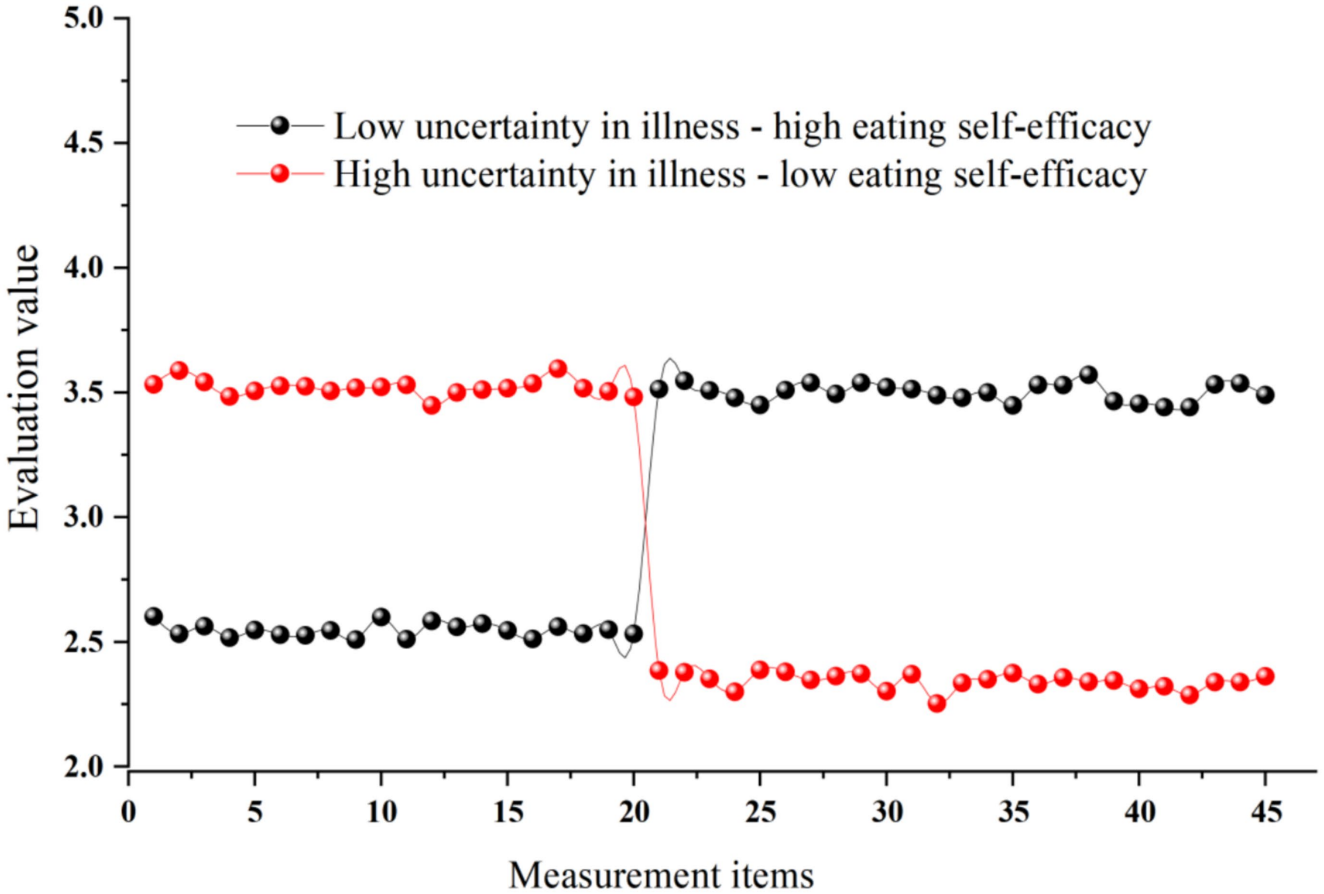
Contour plots of the potential profiles of illness uncertainty and dietary self-efficacy.

Based on the selected solution, patients were categorized into two latent profiles with distinct psychological patterns. Class 1 included 227 participants (53.20%), characterized by lower uncertainty in illness and higher eating self-efficacy, and was labeled the “Low Uncertainty in Illness–High Eating Self-efficacy” group. Class 2 included 199 participants (46.80%), characterized by higher uncertainty in illness and lower eating self-efficacy, and was labeled the “High Uncertainty in Illness–Low Eating Self-efficacy” group. The profile patterns are illustrated in Figure 1, demonstrating a clear crossover pattern and supporting heterogeneous joint distributions of these two psychological constructs among gastric cancer patients.

### 3.4 Univariable comparisons between latent profiles

To examine differences across profiles, chi-square tests were used for categorical variables and independent-samples t tests for continuous variables (Table 4).

**Table 4:** Univariable comparisons of sociodemographic, clinical, lifestyle, and psychological variables between latent profiles.

Variables	Items	Class 1	Class 2	χ^2^/t	p
Gender	Male	105	120	8.395	0.004
	Female	122	79		
Education background	Primary school and below	100	105	6.240	0.044
	Junior high school to high school	90	56		
	Bachelor’s degree or above	37	38		
Marital status	Divorced	16	20	1.282	0.733
	Widowed	21	19		
	Unmarried	24	20		
	Married	166	140		
Employment status	Unemployment	22	25	7.985	0.046
	Retirement	63	74		
	Student	13	5		
	Employed	129	95		
Monthly income level	≤4,000 RMB	80	97	7.988	0.018
	4,001–8,000 RMB	95	67		
	8,001 RMB and above	52	35		
Place of residence	Urban	108	117	5.354	0.021
	Rural area	119	82		
TNM stage	Stage I	82	50	7.596	0.022
	Stage II	80	93		
	Stage III	65	56		
Smoking	Yes.	138	143	5.784	0.016
	No.	89	56		
Drinking alcohol	Yes.	190	151	4.061	0.044
	No.	37	48		
Age	M ± SD	51.30 ± 17.88	52.78 ± 19.943	−0.808	0.420
Health information avoidance	M ± SD	3.049 ± 0.945	3.513 ± 0.769	−5.502	<0.001
Interoceptive sensitivity	M ± SD	2.820 ± 0.775	3.151 ± 0.858	−4.178	<0.001

Class 1: Low uncertainty in illness–high eating self-efficacy group; Class 2: High uncertainty in illness–low eating self-efficacy group.

Significant differences were observed between profiles in gender (χ^2^ = 8.395, *p* = 0.004), education background (χ^2^ = 6.240, *p* = 0.044), employment status (χ^2^ = 7.985, *p* = 0.046), monthly income (χ^2^ = 7.988, *p* = 0.018) and place of residence (χ^2^ = 5.354, *p* = 0.021). But marital status (χ^2^ = 1.282, *p* = 0.733) and age (*t* = −0.808, *p* = 0.420) did not differ significantly. Clinically, TNM stage (χ^2^ = 7.596, *p* = 0.022), smoking history (χ^2^ = 5.784, *p* = 0.016) and alochol (χ^2^ = 4.061, *p* = 0.044) differed significantly.

For psychological variables, the High Uncertainty in Illness–Low Eating Self-efficacy group reported significantly higher health information avoidance (*t* = −5.502, *p* < 0.001) and higher interoceptive sensitivity (*t* = −4.178, *p* < 0.001) than the Low Uncertainty in Illness–High Eating Self-efficacy group.

### 3.5 Multivariable logistic regression

Variables that were significant in univariable analyses were entered into a multivariable logistic regression model to identify independent predictors of profile membership. Using Class 2 (High Uncertainty in Illness–Low Eating Self-efficacy) as the reference category, the model estimated predictors of membership in Class 1 (Low Uncertainty in Illness–High Eating Self-efficacy). Results are shown in Table 5.

**Table 5:** Multivariable logistic regression predicting latent profile membership (Reference Class 2).

Variables	Items	*B*	SE	Wald **χ**^**2**^	*p*	OR	LLCI	ULCI
Health information avoidance		−0.530	0.129	16.893	< 0.001	0.589	0.457	0.758
Interoceptive sensitivity		−0.335	0.138	5.916	0.015	0.715	0.546	0.937
Gender	Male	−0.558	0.219	6.474	0.011	0.572	0.372	0.880
	Female (refer)							
Education background	Primary school and below	−0.103	0.306	0.113	0.736	0.902	0.495	1.644
	Junior high school to high school	0.321	0.324	0.983	0.321	1.379	0.731	2.600
	Bachelor’s degree or above (refer)							
Employment status	Unemployment	−0.560	0.362	2.396	0.122	0.571	0.281	1.161
	Retirement	−0.574	0.247	5.410	0.02	0.563	0.347	0.914
	Student	0.810	0.606	1.787	0.181	2.249	0.685	7.377
	Employed (refer)							
Monthly income level	≤4,000 RMB	−0.686	0.296	5.378	0.02	0.504	0.282	0.899
	4,001–8,000 RMB	−0.146	0.298	0.24	0.624	0.864	0.482	1.549
	8,001 RMB and above (refer)							
Place of residence	Urban	−0.445	0.219	4.109	0.043	0.641	0.417	0.985
	Rural area (refer)							
TNM stage	Stage I	0.429	0.288	2.224	0.136	1.536	0.874	2.699
	Stage II	−0.333	0.266	1.569	0.210	0.717	0.426	1.207
	Stage III (refer)							
Smoking	Yes.	−0.435	0.233	3.480	0.062	0.647	0.41	1.022
	No. (refer)							
Drinking alcohol	Yes.	0.672	0.277	5.873	0.015	1.958	1.137	3.373
	No. (refer)							

OR = odds ratio; CI = Confidence interval.

Health information avoidance was a significant predictor (*B* = −0.530, *p* < 0.001, OR = 0.589, 95% CI: 0.457–0.758). For each one-unit increase in health information avoidance, the odds of belonging to Class 1 decreased by 41.1%. Interoceptive sensitivity was also significant (*B* = −0.335, *p* = 0.015, OR = 0.715, 95% CI: 0.546–0.937), indicating a 28.5% reduction in the odds of belonging to Class 1 per one-unit increase.

Gender remained significant (*B* = −0.558, *p* = 0.011, OR = 0.572, 95% CI: 0.372–0.880). Using female as the reference, male had 42.8% lower odds of belonging to Class 1, suggesting male were more likely to belong to the High Uncertainty in illness–Low Eating Self-efficacy group. For education, none of the levels differed significantly from the reference category (bachelor’s degree or above) (all *p* > 0.050). For employment status, compared with employed participants, retirees had significantly lower odds of belonging to Class 1 (*B* = −0.574, *p* = 0.020, OR = 0.563, 95% CI: 0.347–0.914), whereas differences for unemployed participants and students were not significant.

Regarding monthly income level, compared with ≥ 8,001 RMB, participants earning ≤ 4,000 RMB had significantly lower odds of belonging to Class 1 (*B* = −0.686, *p* = 0.020, OR = 0.504, 95% CI: 0.282–0.899), while 4,001–8,000 RMB did not differ significantly from the reference. For residence, urban residents had significantly lower odds of belonging to Class 1 compared with rural residents (*B* = −0.445, *p* = 0.043, OR = 0.641, 95% CI: 0.417–0.985).

After controlling for covariates, TNM stage was not a significant independent predictor (stage I: *p* = 0.136; stage II: *p* = 0.210; reference = stage III). Smoking history showed a marginal association but did not reach statistical significance (*B* = −0.435, *p* = 0.062, OR = 0.647, 95% CI: 0.410–1.022). Alcohol use history was a significant predictor (*B* = 0.672, *p* = 0.015, OR = 1.958, 95% CI: 1.137–3.373); compared with those without alcohol use, those with alcohol use had 95.8% higher odds of belonging to Class 1.

## 4 Discussion

### 4.1 Latent profile model of uncertainty in illness and eating self-efficacy

Using LPA, we identified two qualitatively distinct subgroups of patients with gastric cancer characterized by different joint patterns of uncertainty in illness and eating self-efficacy: a low uncertainty in illness–high eating self-efficacy group and a high uncertainty in illness–low eating self-efficacy group. The two profiles were relatively balanced in size (53.20% vs. 46.80%), indicating that nearly half of patients experience the combined psychological burden of elevated uncertainty in illness and diminished eating self-efficacy. Notably, the two-profile structure remained stable in the subscale-level sensitivity analysis. Even when uncertainty in illness and eating self-efficacy were represented by five dimension-level indicators rather than aggregated overall measures, the optimal latent structure still consisted of two classes. This finding suggests that the identified profiles were not merely a by-product of using broad total scores. Instead, the heterogeneity observed in this sample appears to reflect a more global pattern of psychological adaptation versus maladaptation. In other words, the dimensions of illness uncertainty tended to cluster together, and the dimensions of eating self-efficacy also tended to move in parallel, rather than forming multiple cross-cutting subgroup configurations. This pattern may be particularly plausible in gastric cancer, where illness-related ambiguity and eating-related challenges are closely intertwined in everyday adjustment.

Within stress-and-coping framework, uncertainty in illness may indirectly shape coping choices and enactment through its influence on primary and secondary appraisal processes (28). When patients perceive substantial ambiguity regarding illness-related information, such cognitive uncertainty may erode confidence in one’s behavioral capacity (10), thereby undermining eating self-efficacy. The inverse configuration observed across the two profiles provides empirical support for this mechanism. Information-processing theory offers an additional explanatory lens: high uncertainty in illness may reflect difficulties in organizing and integrating illness-related information (51), and the consequent depletion of cognitive resources may spill over into dietary self-management, limiting patients’ ability to capitalize on nutritional guidance to build and consolidate eating self-efficacy.

Notably, we did not identify a transitional profile characterized by moderate uncertainty in illness and moderate eating self-efficacy, which may relate to the distinctive context of gastric cancer. As a life-threatening malignancy, gastric cancer diagnosis and treatment can generate profound psychological impact (52), potentially fostering more polarized response patterns rather than intermediate states. Our findings differ from some LPA studies in other cancer populations that have identified three or more adjustment profiles (53). Such discrepancies may stem from heterogeneity across cancer types in clinical characteristics, treatment modalities, and functional sequelae, underscoring the need to validate these patterns in broader oncology populations.

### 4.2 Determinants of optimal profile membership

Both Health Information Avoidance and Interoceptive sensitivity emerged as significant predictors of latent profile membership. Patients with stronger Health Information Avoidance were more likely to belong to the high uncertainty in illness–low eating self-efficacy profile, a finding with clear theoretical implications. Health Information Avoidance reflects motivational tendencies to avoid health-threatening information (54). When patients habitually avoid illness-related information, they are less able to acquire sufficient knowledge to understand disease trajectory, treatment options, and rehabilitation requirements; this information deficit can translate directly into heightened cognitive uncertainty. At the same time, information avoidance restricts access to nutritional guidance and dietary self-management knowledge (55), weakening the knowledge base needed to develop eating self-efficacy. This aligns with prior evidence suggesting that information avoidance plays a maladaptive role in psychological adjustment among patients with cancer (56, 57).

Higher Interoceptive sensitivity also predicted membership in the high uncertainty in illness–low eating self-efficacy profile, a result that may initially appear counterintuitive. Interoceptive sensitivity refers to an individual’s capacity to perceive and attend to internal bodily signals (58); in principle, greater interoceptive sensitivity could facilitate monitoring of bodily responses and adaptive dietary adjustments (59). Postoperative patients commonly experience symptoms such as dumping syndrome, reflux, and abdominal distension (60); those with high Interoceptive sensitivity may over-attend to and amplify such sensations, perpetuating worry and uncertainty about illness status.

Male patients were more likely to belong to the high uncertainty in illness–low eating self-efficacy profile. Men may be more inclined to adopt suppressive or avoidant coping styles and may be less likely to seek emotional support or health information proactively (61, 62), contributing to higher uncertainty in illness. In addition, within traditional Chinese cultural contexts, meal preparation and dietary management are often socially ascribed to women; consequently, men may face greater challenges in postoperative dietary adjustment due to limited prior knowledge and skill acquisition, manifesting as lower eating self-efficacy.

Employment status also warranted attention. Compared with employed patients, retirees were more likely to belong to the high uncertainty in illness–low eating self-efficacy profile. This association may reflect characteristics of retirement populations, including older age, greater comorbidity burden, and functional decline, all of which increase the complexity of disease management (63). Retirement may also entail reduced workplace-based social networks, limiting access to health information and social support (64). By contrast, employed individuals may possess more social resources, stronger information-seeking capacity, and a more active life orientation, which may help sustain lower uncertainty in illness and higher eating self-efficacy.

Monthly personal income represented a key socioeconomic determinant of profile membership. Lower-income patients were more likely to belong to the high uncertainty in illness–low eating self-efficacy profile, highlighting the role of socioeconomic status in cancer adjustment. Limited financial resources may constrain access to high-quality healthcare services, nutritional supplements, and professional dietary counseling (65), while heightening concerns regarding treatment costs and quality of life, thereby increasing uncertainty in illness. Additionally, low-income patients may face constrained food choices, making it more difficult to purchase and prepare recommended foods and further weakening eating self-efficacy.

Residence type showed an intriguing pattern: urban residents were more likely than rural residents to belong to the high uncertainty in illness–low eating self-efficacy profile, contrary to some expectations. Urban residency is often assumed to confer better access to medical resources and information channels and thus better psychological adjustment (66). Our findings may instead reflect potential adverse features of urban lifestyles. Urban residents may experience faster-paced living, greater occupational stress, and more complex social demands, increasing psychological burden. Moreover, although urban residents may be exposed to larger volumes of health information, the complexity and inconsistency of such information may paradoxically exacerbate uncertainty in illness. Rural residents, despite relatively fewer medical resources, may benefit from tighter family support networks and simpler daily routines that facilitate adjustment.

The predictive role of alcohol use history also merits careful interpretation. Patients with an alcohol use history were more likely to belong to the low uncertainty in illness–high eating self-efficacy profile. This association may reflect differences in personality traits or coping styles, or residual confounding by socioeconomic status or social support. Importantly, these results should not be construed as endorsing alcohol consumption; alcohol use is a recognized risk factor for gastric cancer and may adversely affect postoperative recovery. Smoking showed a near-significant association with profile membership, suggesting that the relationship between smoking and adjustment typologies may be complex and warrants further investigation.

### 4.3 Practical and clinical implications

The pronounced heterogeneity in uncertainty in illness and eating self-efficacy among patients with gastric cancer indicates that clinicians should move beyond “one-size-fits-all” approaches toward stratified and personalized supportive care. Patients classified into the high uncertainty in illness–low eating self-efficacy profile should be prioritized for more intensive and comprehensive psychological care and nutritional support.

To address elevated uncertainty in illness, clinical teams should strengthen informational support by proactively assessing patients’ information needs and providing clear, comprehensible explanations regarding staging, treatment options, prognosis, and recovery trajectories, thereby helping patients develop a coherent cognitive framework for understanding their illness. Given that Health Information Avoidance is a key predictor of uncertainty in illness, clinicians should assess avoidance tendencies and adopt gradual, non-threatening communication strategies to facilitate engagement with essential health information.

To improve low eating self-efficacy, nutrition teams should deliver structured dietary counseling and skills training, including individualized meal plans, demonstrations of food preparation techniques, recipe guidance, and stepwise goal setting. In practice, peer-support groups can be established in which well-recovered gastric cancer survivors share dietary self-management experiences to provide vicarious learning opportunities. Healthcare professionals should also provide positive feedback and encouragement to reinforce confidence in dietary behavior change.

At admission or during follow-up, brief assessments could flag patients at elevated risk—particularly men, retirees, low-income patients, urban residents, and those with strong Health Information Avoidance—and enable early provision of preventive psychological support and dietary counseling. For patients with high Interoceptive sensitivity, clinicians may consider mind–body strategies such as mindfulness training to cultivate nonjudgmental awareness of bodily sensations and reduce excessive symptom monitoring and catastrophizing.

### 4.4 Limitations and future directions

This study employed a cross-sectional design, which can delineate associations but does not support causal inference. Future work should adopt longitudinal designs with repeated measurements to characterize trajectories over time and clarify reciprocal mechanisms.

Participants were recruited via convenience sampling from two tertiary hospitals in Zhejiang Province, potentially limiting representativeness and generalizability. Future studies should broaden recruitment across regions and healthcare settings to enhance external validity. Additionally, all variables were assessed via self-report questionnaires, raising concerns about common method and social desirability biases. Future research should incorporate multi-source assessments (e.g., clinician-rated evaluations, caregiver reports, objective physiological indicators). For eating self-efficacy, integrating dietary diaries and objective nutritional status indicators would help validate self-reported measures.

Although the predictors included demographic, clinical, lifestyle, and psychological domains, other influential factors may have been omitted, such as social support, coping styles, quality of patient–clinician communication, and prior mental health status. Future studies could extend the predictor set to build more comprehensive models. Moreover, we did not stratify patients by treatment phase (e.g., preoperative, early postoperative, recovery period), despite likely phase-specific differences in psychological status; targeted phase-based investigations are warranted.

From a methodological standpoint, the primary LPA employed 45 item-level indicators, which resulted in a relatively large number of freely estimated parameters relative to the sample size. Although the sample-to-parameter ratio for the two-class model was modest, several factors mitigate concerns about estimation reliability, including the high entropy value, balanced class proportions, successful replication of the best log-likelihood across multiple random starts, and—most critically—the convergent results obtained from a sensitivity analysis using five subscale-level indicators with a substantially more favorable sample-to-parameter ratio. Nonetheless, future studies with larger samples could further strengthen confidence in item-level LPA solutions and potentially support the identification of more fine-grained latent profiles. Additionally, alternative approaches such as factor mixture modeling or the use of parceled indicators may offer complementary strategies for balancing model complexity with sample size constraints.

Finally, while the two-profile solution exhibited high classification precision, it did not further differentiate more granular subgroups. This may reflect the sample size or the selection of indicators. Larger samples and inclusion of additional adaptation-relevant variables (e.g., anxiety, depression, quality of life) may reveal more nuanced latent structures and enable a more comprehensive typology of psychological characteristics in gastric cancer.

## 5 Conclusion

This study identified two latent profiles of uncertainty in illness and eating self-efficacy among patients with gastric cancer: a low uncertainty in illness–high eating self-efficacy profile and a high uncertainty in illness–low eating self-efficacy profile. Health information avoidance, interoceptive sensitivity, sex, employment status, income, residence, and alcohol use history were associated with profile membership. These findings highlight psychological heterogeneity in gastric cancer and may help inform more individualized supportive care.

## Data Availability

The raw data supporting the conclusions of this article will be made available by the authors, without undue reservation.
